# Effect of long-term high-fat diet intake on peripheral insulin sensibility, blood pressure, and renal function in female rats

**DOI:** 10.3402/fnr.v60.28536

**Published:** 2016-02-12

**Authors:** Noemi A. V. Roza, Luiz F. Possignolo, Adrianne C. Palanch, José A. R. Gontijo

**Affiliations:** Laboratório de Metabolismo Hidrossalino, Núcleo de Medicina e Cirurgia Experimental, Departamento de Clínica Médica, Faculdade de Ciências Médicas, Universidade Estadual de Campinas, Campinas, Brazil

**Keywords:** blood pressure, glucose tolerance test, high-fat diet, insulin sensitivity, renal function

## Abstract

**Background:**

This study determines whether 8-week high-fat diet (HFD) consumption alters insulin sensitivity, kidney function, and blood pressure (BP) in female rats when compared with standard rodent diet (ND) intake in gender- and age-matched rats.

**Methods:**

The present study investigates, in female Wistar HanUnib rats, the effect of long-term high-fat fed group (HFD) compared with standard chow on BP by an indirect tail-cuff method using an electrosphygmomanometer, insulin and glucose function, and kidney function by creatinine and lithium clearances.

**Results:**

The current study shows glucose tolerance impairment, as demonstrated by increased fasting blood glucose (ND: 78±2.8 vs. HFD: 87±3.8 mg/dL) associated with reduced insulin secretion (ND: 0.58±0.07 vs. HFD: 0.40±0.03 ng/mL) in 8-week female HFD-treated rats. The incremental area under the curve (AUC, ND: 1,4558.0±536.0 vs. HFD: 1,6507.8±661.9), homeostasis model assessment of insulin resistance (HOMA-IR) index, and the first-order rate constant for the disappearance of glucose (*Kitt*) were significantly enhanced in 8-week HFD-treated rats compared with age-matched ND group (respectively, *P*=0.03, *P*=0.002, and *P*<0.0001). The current study also shows a significantly higher systolic BP measured in 5 and 8 weeks posttreatment in HFD (5-week HFD-treated: 155.25±10.54 mmHg and 8-week HFD-treated: 165±5.8 mmHg) (*P*=0.0001), when compared to BP values in 5-week ND, 137±4.24 mmHg and 8-week ND, 131.75±5.8 mmHg age-matched group. Otherwise, the glomerular filtration rate and renal sodium handling evaluated by FE_Na_, FEP_Na_ and FEPP_Na_, were unchanged in both groups.

**Conclusion:**

We may conclude that 8-week female HFD-fed rats compared with ND group stimulate harmful effects, such as BP rise and peripheral glucose intolerance. The increased BP occurs through insulin resistance and supposedly decreased vasodilatation response without any change on renal function.

The incidence of obesity and cardiovascular disease is strongly associated with the increased ingestion of caloric foods, as characterized by the western dietary pattern. Over the last decades, changes in the Brazilian diet have revealed a high consumption of simple sugars, saturated and *trans-*fats and a reduction in the ingestion of vegetables, fruits, and fibers (1, 2). In the United States, approximately two-thirds of adults are overweight or obese (3).

Globally, cardiovascular diseases are the leading cause of death and could be avoided with a healthy and balanced diet (4). Also, obesity increases the risk of kidney disease and hypertension by fourfold and accounts for 25% of all chronic renal failure patients. Additionally, the risk of developing insulin resistance is enhanced when obesity is combined with high blood pressure (BP) and metabolic disorders (5–8). Recently, reports suggested that metabolic syndrome is related to the increased risk for developing chronic kidney diseases (5, 9–12) and renal lipid accumulation associated with lipotoxicity (13, 14).

The high-fat diet (HFD) induces changes in renal lipid metabolism due to an imbalance between lipogenesis and lipolysis in the kidneys, as well as systemic metabolic abnormalities and subsequent renal lipid accumulation and renal injury (10, 15). The glomerular and tubulointerstitial lesions (16–18) associated with chronic glomerulopathy (13, 18), nephrotic syndrome (19), chronic renal failure (12), diabetic nephropathy (20), obesity-associated renal disease (10), and aging nephrosclerosis (21, 22) are related to renal tissue lipid accumulation. In rats, the HFD causes renal injury preceded by endothelial dysfunction and hypertension, both induced by increased oxidative stress, an exacerbated inflammatory response, and disruption of the renal filtration barrier (11, 18, 22).

Taking into account the above findings, the purpose of the present study was to determine whether the long-term HFD intake alters insulin sensitivity, kidney function, and arterial BP in female rats compared with a gender- and age-matched control group (ND) fed standard rodent chow. Since the long-term changes in renal sodium tubule handling are associated with hypertensive development, we also hypothesized that HFD intake may cause a decrease in urinary sodium excretion in the experimental group. To test this hypothesis, we studied the tubular sodium handling, evaluated by lithium clearance (CLi), in conscious HFD female rats and compared with their appropriate standard diet controls.

## Material and methods

### Animals

The experiments were conducted on age-matched rats of sibling-mated Wistar HanUnib rats allowed free access to water and normal rat chow. The general guidelines established by the Brazilian College of Animal Experimentation (COBEA) and approved by the Institutional Ethics Committee (#1697-1) were followed throughout the investigation. Our local colonies originated from a breeding stock supplied by CEMIB/Unicamp, Campinas, SP, Brazil. Immediately after weaning, at 3 weeks of age (60.7±1.3 g body weight), female Wistar rats were housed in individual cages and maintained under controlled temperature (22°C) and lighting conditions (07:00 h–19:00 h), with free access to tap water. These animals were randomly distributed into two dietary groups: the control group was fed standard pelleted rodent chow (ND) (Nuvital, Curitiba, PR, Brazil); the HFD group was fed an HFD (HFD AIN-93G)-modified diet as recommended to support growth, pregnancy, and lactation phases by American Institute of Nutrition, 1993 (see Table 1) (23) and followed up to 11 weeks of age. The standard chow diet contained 15.86% kcal as fat and a total of 3.97 kcalories/g, and the HFD contained 60.57% kcal as fat and a total of 5.45 kcalories/g (Table 1). All females were weighed weekly and had food and water intakes measured daily throughout experiment.

**Table 1:** The table shows the composition of pelleted standard rodent laboratory chow and of long-term high-fat diet (HFD)

Ingredients	Standard (g/kg)	HF diet 60% (g/kg)
Amido	397.5	52
Corn starch dextrinizade	132	143
Sucrose	100	66
*Carbohydrate*	*629.5*	*261*
Casein	200	271
L-Cysteine	3	3
Choline bitartrate	2.5	2.5
*Protein*	*205.5*	*276.5*
Soybean oil	70	34
Lard (saturated fat)	–	333
*Total fats*	*70*	*367*
Cellulose microfine	50	50
*Fiber*	*50*	*50*
Mineral mix	35	35
Vitamin mix	10	10
*Energy content*	3.97 kcalories/g	5.45 kcalories/g

AIN 93G modified as recommended by American Institute of Nutrition, 1993) (23). The standard chow contained 15.86% kcal as fat and 3.97 kcalories/g of chow, while the high-fat diet contained 60.57% kcal as fat and a total of 5.45 kcalories/g of chow.

### BP measurement

The systolic blood pressure (SBP) was measured in conscious ND and HFD rats after 5 and 8 weeks of standard or HFD treatment, employing an indirect tail-cuff method using an electrosphygmomanometer combined with a pneumatic pulse transducer/amplifier (IITC Life Science – BpMonWin Monitor Version 1.33). This indirect approach allowed repeated measurements with a close correlation (correlation coefficient=0.975), compared with direct intra-arterial recording (24–26). The mean of three consecutive readings represented the BP.

### Renal function test

The renal function tests were performed after 8 weeks of treatment with ND or HFD in conscious, unrestrained rats. Briefly, 14 h before the renal test, 60 µmol LiCl 100 g^−1^ body weight was given by gavage. After an overnight fast, each animal received a load of tap water by gavage (5% of body weight), followed by a second load of the same volume, 1 h later, and spontaneously, voided urine was collected over a 120-min period into a graduated centrifuge tube. At the end of all the renal function test experiments, blood samples were drawn by tail vein puncture in anesthetized rats, and urine and plasma samples were collected and immediately stored at −20°C until processing. The proteinuria was detected using the Sensiprot Kit (Labtest). The creatinine clearance (CCr) used to estimate the glomerular filtration rate and the CLi used to estimate the sodium output from the proximal tubule were calculated by standard formula ((U.V)/P), where U is the urinary creatinine and lithium concentrations, V is the urinary flow, and P is the creatinine and lithium plasma levels. Fractional sodium (FE_Na_) and potassium (FE_K_) excretion were calculated as C_Na_/C_Cr_×100 and CE_K_ */*CF_K_×100, respectively, where C_Na_ is the sodium clearance, CE_K_ is the potassium clearance, CCr is the creatinine clearance, and CF_K_ is the filtered load potassium. The fractional proximal (FEP_Na_) and post-proximal (FEPP_Na_) sodium excretion were calculated as C_Li_/C_Cr_×100 and C_Na_ */*C_Li_×100, respectively (24–26).

### Glucose tolerance test

The glucose tolerance test (GTT) was performed after 8 weeks of treatment and 12 h of fasting in order to determine changes in insulin sensitivity. Eleven rats from independent litters were tested. To establish basal values of glucose and insulin, blood samples were taken by lancing the tail vein before glucose challenge (time 0). Then, they received a single bolus of 1 g/kg glucose i.p. Blood samples were taken at 15, 30, 60, 90, and 120 min from the tail vein. Plasma was separated (50 µl) and kept at −20°C for measurement of insulin levels by radioimmunoassay. The incremental area under the glucose tolerance curve (AUC) was calculated as the integrated area under the curve above the basal value (time 0) over the 120-min sampling period using Prism 4 for Windows.

### Insulin tolerance test

Insulin tolerance tests were performed after 8 weeks of treatment and 6 h of fasting in order to determine changes in peripheral insulin sensitivity. Fifteen rats from independent litters were tested. To establish basal values of glucose and insulin, blood samples were taken by lancing the tail vein before insulin challenge (time 0). They then received a single bolus of 0.67 UI/g of body weight insulin i.p. Blood samples were taken at 5, 10, 15, 20, 25, and 30 min from the tail vein. Thereafter, the rate constant for plasma glucose disappearance (*Kitt*) was calculated using the formula 0.693/*t*_1/2_. The plasma glucose *t*_1/2_ was calculated from the slope of the least squares analysis of the plasma glucose levels during the linear phase of decline curve. On the basis of fasting plasma, an insulin and glucose levels homeostasis model assessment of insulin resistance (HOMA-IR) index was calculated according to the formula: HOMA-IR=fasting insulin (ng/mL)×fasting blood glucose (mg/dL)/405 (5).

### Biochemical analysis

Plasma and urine sodium, potassium, and lithium concentrations were measured by flame photometry (Micronal, B262, São Paulo, Brazil), while creatinine concentrations were determined spectrophotometrically (Instruments Laboratory, Genesys V, USA). Glucose from whole blood was measured with a glucometer MediSense/Optium, Abbott. Plasma concentrations of insulin were measured by immunoassay (Millipore, Billerica, USA, with sensibility of 0.05 ng/mL). The plasma samples for urea, albumin, globulin, total protein, chloride, magnesium, calcium, phosphorus, HDL cholesterol, LDL cholesterol, and triglyceride levels were also collected on the 8th week posttreatment in ND and HFD and measured by a Modular Analytic P Biochemistry Analyzer (Roche^®^) according to the manufacturers’ protocols for clinical and immunochemistry assays.

### Data presentation and statistics

Data obtained from this study are expressed as the mean±SEM. Data obtained over time were analyzed using a repeated measures two-way ANOVA test. *Post hoc* comparisons between selected means were performed with Bonferroni's contrast test when initial two-way ANOVA indicated statistical differences between experimental groups. Comparisons involving only two means within or between groups were carried out using a Student's *t -*test. The level of significance was set at *P*≤0.05.

## Results

### Experimental model data

Tables 2 and 3 present the plasma and urine biochemical levels from 8-week female ND (*n*=21) and HFD-treated (*n*=21) groups. There were no significant differences between plasma biochemical parameter levels in female rats obtained after 8 weeks of HFD treatment compared with appropriated gender- and age-matched control (ND) group. Table 3 shows the parameters from the urine biochemical analysis in the same groups. The urine potassium, calcium, phosphorus, uric acid, amylase, and creatinine levels were similar in both experimental groups. However, the urine levels of sodium (4.7±0.4 mEq/L vs. 6.2±0.4 mEq/L) and chloride (7.1±0.3 mEq/L vs. 9.0±0.4 mEq/L) were increased in HFD group, while the magnesium (1.9±0.1 mEq/L vs. 1.3±0.1 mEq/L) and urea (276.7±21.7 mg/dL versus 211.8±11.4 mg/dL) levels were significantly lower in HFD compared with ND age-matched rats.

**Table 2:** The table shows the plasma biochemical parameter levels from 8-week female HFD-treated rats (*n*=10) compared with appropriated gender- and age-matched controls (ND; *n*=10)

	ND	HFD
Sodium (mEq/L)	141.7±0.9	141.7±1.0
Potassium (mEq/L)	4.3±0.2	4.3±0.3
Chloride (mEq/L)	104.3±0.4	106.0±1.2
Magnesium (mEq/L)	1.9±0.044	1.7±0.044
Calcium (mg/dL)	9.3±0.2	9.4±0.2
Phosphorus (mg/dL)	6.8±0.4	6.7±0.3
Cholesterol (mg/dL)	50.6±3.5	49.3±5.1
Triglycerides (mg/dL)	37.4±3.5	40.0±1.6
HDL cholesterol (mg/dL)	47.0±3.1	46.3±4.6
LDL cholesterol (mg/dL)	6.3±0.5	4.8±0.5
VLDL (mg/dL)	8.8±1.3	6.8±1.2
Glucose (mg/dL)	80.0±3.0	92.0±5.0*
Urea (mg/dL)	39.0±2.0	40.8±2.3
Creatinine (mg/dL)	0.4±0.005	0.4±0.1
Albumin (g/dL)	4.0±0.1	4.1±0.1
Globulin	2.7±0.6	2.0±0.0
Total protein (g/dL)	6.0±0.1	5.8±0.1

Results are expressed as means±SEM.

* *P*≤0.05 versus control (Student's *t*-test).

**Table 3:** The table shows the urinary biochemical parameter levels in isolated samples from 8-week female HFD-treated rats (*n*=10) compared with appropriated gender- and age-matched controls (ND; *n*=10)

	ND	HFD
Sodium (mEq/L)	4.7±0.4	6.2±0.4*
Potassium (mEq/L)	3.6±0.4	3.1±0.4
Chloride (mEq/L)	7.1±0.3	9.0±0.4**
Magnesium (mEq/L)	1.9±0.1	1.3±0.1**
Calcium (mg/dL)	0.5±0.1	0.6±0.1
Phosphorus (mg/dL)	1.7±0.6	1.8±0.5
Uric Acid (mg/dL)	0.2±0.046	0.1±0.048
Amylase (U/L)	1.0±0.7	2.2±1.6
Urea (mg/dL)	276.7±21.7	211.8±11.4*
Creatinine (mg/dL)	3.4±0.4	3.7±0.5

Values are means±SEM.

* *P*<0.05;

** *P*<0.01 (Student's *t*-test).

**Fig. 1:**
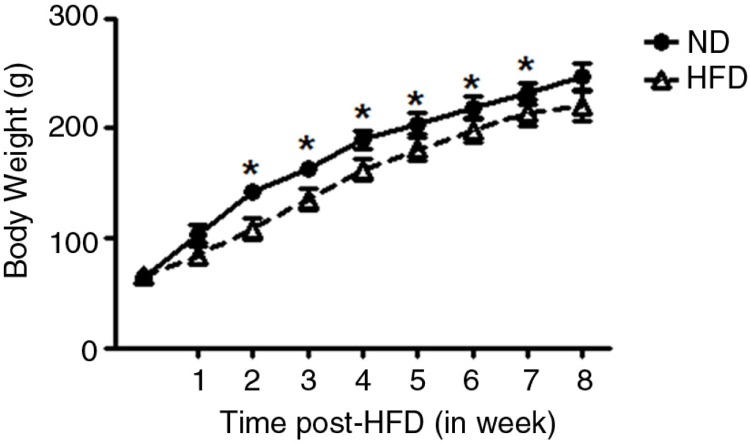
The figure shows body weight (in grams) of female rats obtained throughout 8 weeks of HFD (*n*=21) treatment compared with appropriated gender- and age-matched controls (ND) (*n*=21). Results are expressed as means±SEM. *P≤0.05 or **P≤0.01 versus ND (two-way ANOVA; *post hoc* Bonferroni's contrast test).

**Fig. 2:**
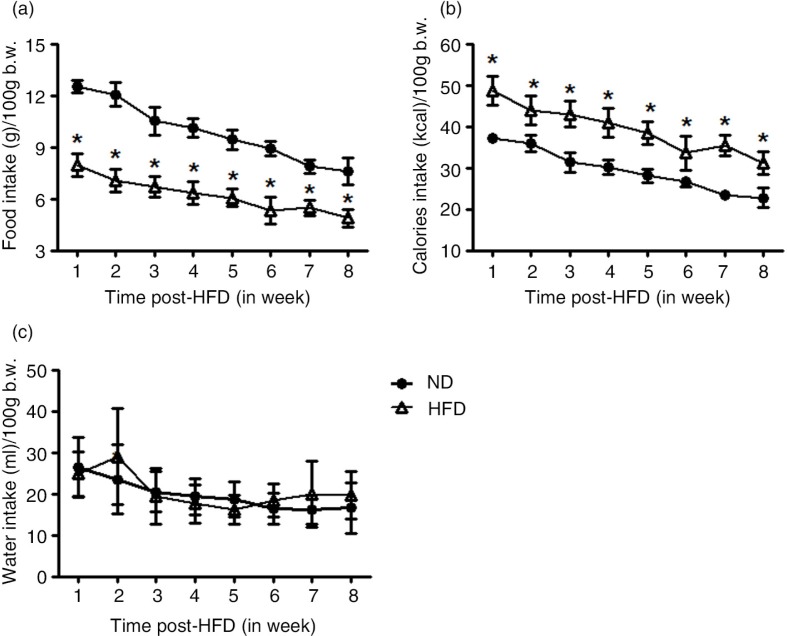
Graphical representation depicts food intake (g/100 g b.w.) (panel a); caloric intake (kcalories/100 g b.w.) (panel b); and water intake (mL/100 g b.w.) (panel c) throughout 8 weeks of standard (ND or CTL, *n*=9) and high-fat diet (HFD, *n*=9) consumption. Data are expressed as means±SEM. *P≤0.05 or **P≤0.01 versus ND (two-way ANOVA; *post hoc* Bonferroni's contrast test).

**Fig. 3:**
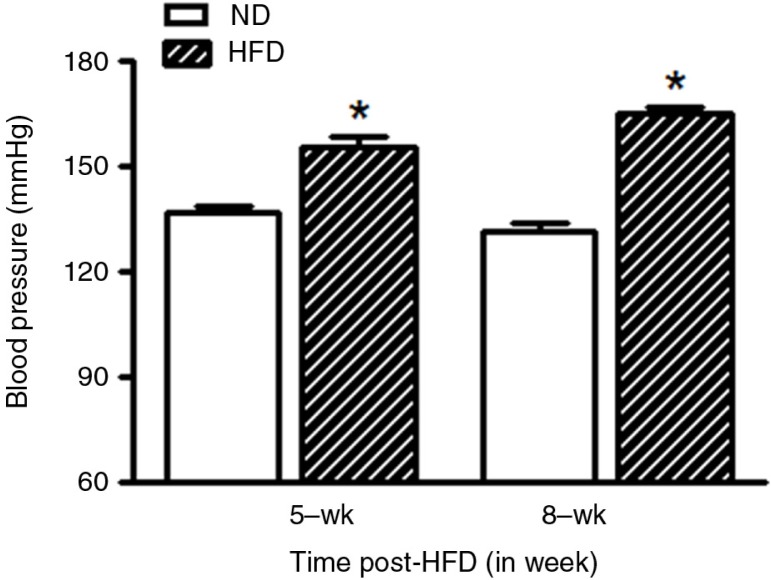
Graphical representation of arterial systolic blood pressure (mmHg) measured in conscious female HFD (*n*=9) rats compared with gender- and age-matched ND (*n*=9) group after 5 and 8 weeks of standard or high-fat diet treatment. Values are means±SEM. *P<0.05 versus ND (Student's *t*-test).

As shown in Fig. 1, the initial body mass was not different between all groups. However, HFD grew less rapidly over the experimental period, and significant differences were observed after the 2nd week of HFD treatment, when compared with ND group (*P*<0.05). The food, calorie energy consumption, and water intake data are presented in Fig. 2. The food and calorie intake, and water consumption were, respectively, expressed in grams (Fig. 2a), kcalories (Fig. 2b), and mL (Fig. 2c) per 100 g of body weight. In general, food intake and therefore sodium intake were reduced significantly in HFD female rats between the 1st and 8th weeks of treatment (*P*<0.04) during follow-up, when normalized by body weight; however, the energy consumption was increased in HFD, when compared with ND animals. The water ingestion was not different between both groups, throughout the 8 weeks of follow-up (Fig. 2c). Figure 3 shows a significantly higher SBP (in mmHg) measured at 5 and 8 weeks posttreatment in HFD (5-week HFD-treated: 155.25±10.54 mmHg and 8-week HFD-treated: 165±5.8 mmHg) (*P*=0.0001), when compared with BP values at 5-week ND (137±4.24 mmHg) and 8-week ND (131.75±5.8 mmHg) age-matched control group.

### Fasting glucose and insulin levels and GTT

**Fig. 4:**
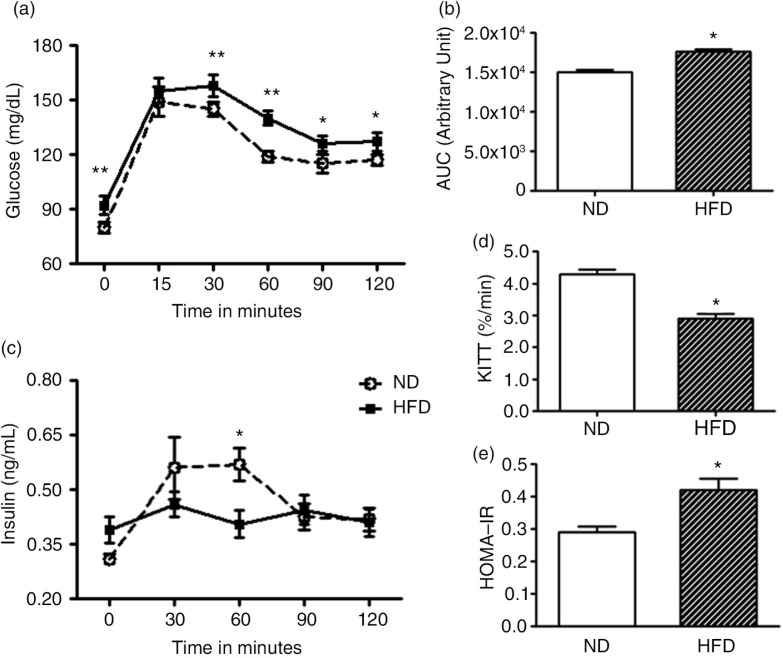
Graphical representation depicts glucose curve (mg/dL) (panel a) after overnight fasting; the incremental AUC (panel b); and insulin plasma levels (ng/mL) (panel c) at different time points (0, 15, 30, 60, 90, and 120 min) during GTT or insulin tolerance test (*Kitt*) (panel d); and homeostasis model assessment of insulin resistance (HOMA-IR) index (panel e) after 8 weeks of ND (*n*=10) or HFD (*n*=10) treatment. Values are means±SEM. *N*=15 for each group; *P<0.05 (Student's *t*-test).

The fasting glucose and insulin levels and GTTs were performed to verify the effect of HFD (*n*=10) treatment on glucose tolerance, compared with the ND (*n*=10) group (Fig. 4). The current study shows that fasting glucose plasma level was significantly increased in female HFD when compared with age-matched ND rats (*P*<0.024) (for details see Table 2, Fig. 4a). Otherwise, after glucose intraperitoneal loading, the HFD group achieved significantly higher plasma glucose concentrations than the ND group at 30, 60, 90, and 120 min (Fig. 4a). Thus, the incremental AUC in HFD was significantly higher (*P*<0.0001) compared with the ND group (Fig. 4b). The study shows that the HFD group (HFD: 0.42±0.07 ng/mL) showed higher basal plasma insulin after 6-h fasting, when compared with the ND (ND: 0.29±0.03 ng/mL) age-matched rats (*P*<0.03) (Fig. 4c). Otherwise, the first-order rate constant for the disappearance of glucose (*Kitt*) over the period 5–30 min, taken also as a measure of insulin sensitivity, was significantly decreased in HFD-treated rats compared with ND group (*P*=0.002) (Fig. 4d). Also, the homeostasis model assessment of insulin resistance (HOMA-IR) index, taken as a measure of insulin resistance, was significantly enhanced in 8-week female HFD-treated rats compared with age-matched ND group (*P*=0.003) (Fig. 4e).

### Renal function test

**Fig. 5:**
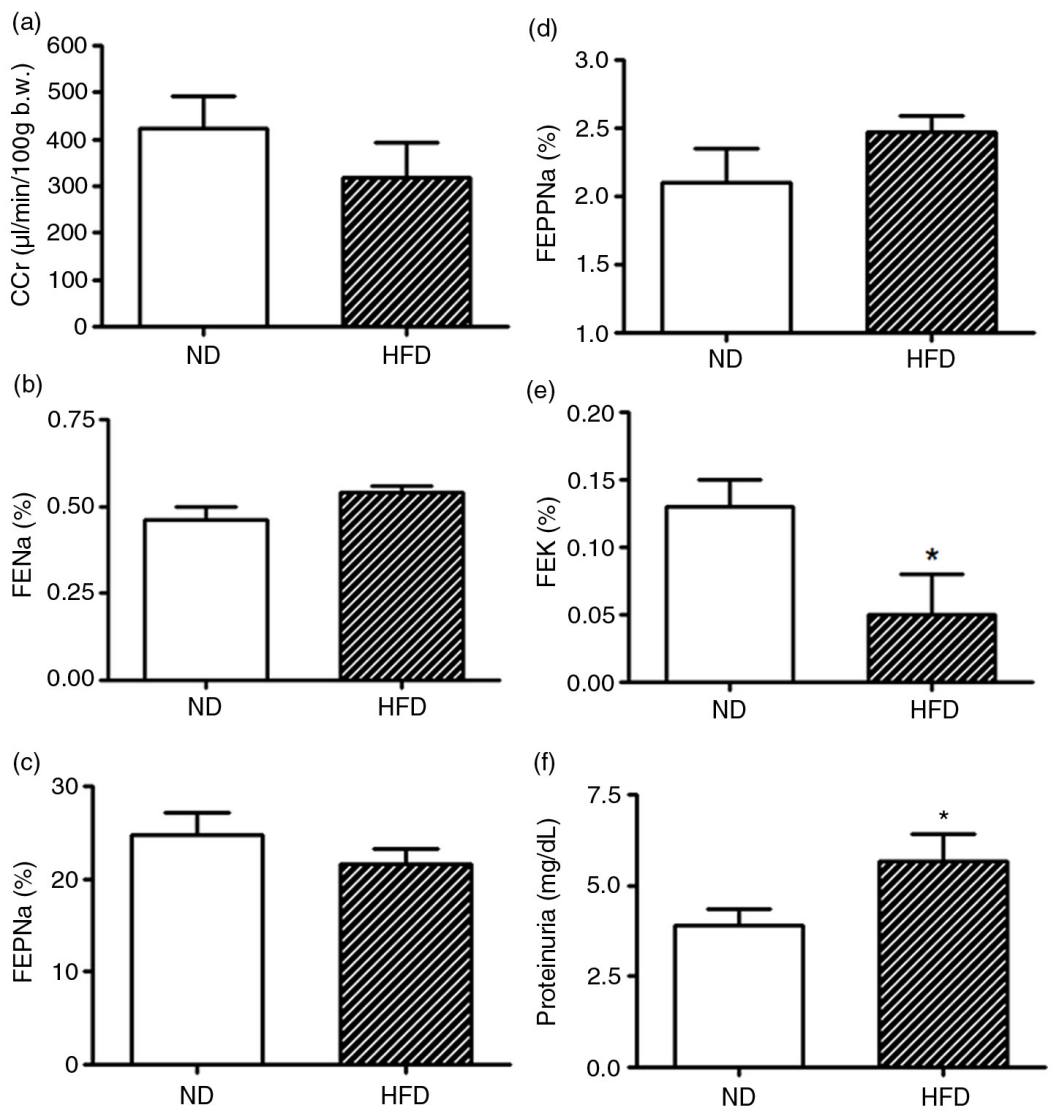
Renal function studies of the creatinine clearance (C_Cr_, panel a), fractional sodium excretion (FE_Na+_, panel b), proximal (FEP_Na+_, panel C), and post-proximal (FEPP_Na+_, panel d), fractional sodium excretion and fractional potassium excretion (FE_K+_, panel e), and urinary protein excretion (panel f) in female rats after 8 weeks of HFD treatment compared with appropriated age-matched controls (ND) (*n*=10 for each group). Results are expressed as means±SEM. *P ≤ 0.05 versus ND (Student's *t-*test).

The data from renal function tests in the 8-week ND (*n*=10) and HFD-treated (*n*=10) rats are summarized in Fig. 5. The urinary flow rate (data not included) was significantly lower in the HFD when compared with the ND group during the renal tubule sodium handling study. Otherwise, the glomerular filtration rate and renal sodium handling evaluated by FE_Na_, FEP_Na_, and FEPP_Na_, estimated by C_Cr_, and CLi, were unchanged in long-term HFD rats, when compared with the ND group. In the present study, the renal test study shows that 8-week HFD-treated rats excreted less potassium than the age-matched ND rats. This is further highlighted by the significant differences in fractional potassium excretion during the same time period (Fig. 5). The significantly decreased kaliuresis effect, observed in HFD rats (0.10±0.01%), was unaltered in the control group of rats (0.15±0.02%). Despite unchanged renal function was observed a significant increase in the urinary protein excretion in 8-week HFD-treated rats when compared with appropriated ND group (Fig. 5f).

## Discussion

More and more evidence is emerging that highlights the far-reaching consequences of obesity and HFD on metabolic and cardiovascular disorders. Driving forces for overweight and obesity include increasing sedentary lifestyles and consumption of a western diet high in fat, fructose, and salt and their interaction with genetic factors and epigenetic processes (6–8). The present study found an impairment of glucose tolerance, as demonstrated by increased fasting blood glucose level and increased blood glucose level (also expressed by an incremental area under the glucose curve) accompanied by decreased constant for the disappearance of glucose (*Kitt*), higher HOMA-IR index, and reduced insulin secretion during a GTT in 8-week HFD-treated female rats. These findings strongly suggest the occurrence of insulin resistance and, as previously demonstrated in metabolic syndrome models, a defect in insulin-stimulated glucose uptake (5–8). This study also shows that the insulin secretion during the first 30 min after glucose was nearly halved in HFD rats. Thus, the current study demonstrates that female Wistar rats fed on an HFD for 8 weeks exhibited some of the hallmark features of the diet-induced metabolic syndrome model, such as mild hyperglycemia, reduced insulin secretion, and moderately increased SBP when compared with same parameters studied in standard ND-fed animals.

The present experimental model of the female HFD-fed rats promotes glucose intolerance, as suggested by hyperglycemia, associated with normal insulinemia levels. Supporting the present findings, the study by Ng et al. (27) examined pancreatic islets and β-cell abundance and performed a genome-wide microarray analysis of isolated islets to explore the mechanisms of impaired insulin secretion. In that study, the HFD animals showed a reduced relative islet area, mainly owing to reduced large islets, and tended to have a reduced β-cell area, implying impaired β-cell replication. Taking into account the present and previous study, we may propose that a limited β-cell reserve in the female HFD-treated rats is sufficient to maintain fasting glucose and insulin levels close to normal, but is inadequate to preserve glucose-stimulated insulin secretion and glucose tolerance.

Unexpectedly, during the follow-up period of time, the female HFD-treated animals showed a decrease in the body weight, and food and water intake, despite greater caloric intake, beyond 5th week of age when compared with age-matched ND rats. Interestingly, the decreased body mass under conditions of high caloric intake and high-fat diet availability occurred in the absence of overfeeding, suggesting that these adaptive responses were mainly driven by the macronutrient composition of the diet. Additionally, this study was not able to show any change in adiposity and fasting plasma triglyceride or cholesterol concentrations in adult female rats after 8 weeks of HFD treatment when compared with ND group. In this way, current data are coincident with recent study showing that HFD in rats induces elevation of BP, heart rates, and higher increased visceral lipid stores, constituting the best nutritional interventions to induce metabolic syndrome in rats (28). However, in this study, our findings did not demonstrate changes in fasting plasma triglyceride and cholesterol in adult HFD rats compared with age-matched control group. In the present study, we may suppose that lowest body weight and intake of food could be related to known decreased muscle mass, satiety, as well a slow gastric emptying induced by long-term high-fat saturated diet ingestion. We may also suggest that either obesity may emerge later or it may not progress through the Wistar lineage in rodents, as reported by studies on undernourished (28) and HFD-fed (18) animals.

Previous studies have demonstrated specific effects of the age, obesity, HFD, and gender on insulin sensitivity and skeletal muscle mitochondrial function (29, 30). Briefly, they show that a higher mitochondrial ATP production capacity was noted in the men, whereas the women were more insulin-sensitive, demonstrating further dissociation between insulin sensitivity and muscle mitochondrial function. Estrany et al. (30) supporting the present study show that HFD feeding did promote an increase in adiposity, although only in male rats. Reciprocal to the present study, however, HFD impaired glucose tolerance and insulin sensitivity markers in adipose tissue of male rats, but not in female rats. These studies suggest that male rats seem to be more prone to disorders associated with an unbalanced composition of the diet, even in the absence of hyperphagia. In contrast, female rats counteract excessive fat intake by improving their ability to use lipid fuels, which limits adiposity but, as demonstrated in our study, is associated with impaired glucose tolerance and insulin sensitivity. As can be seen in our and other studies cited above, the metabolic disorders induced by the ingestion of HFD are still contradictory to poorly understood intrinsic pathophysiological mechanisms.

Here, with the data from 8-week female HFD-treated rats, it has been supposed that insulin resistance may result from a cluster of metabolic disorders with an inherent potential for hemodynamic abnormalities, particularly, arterial hypertension, when compared with age-matched ND intake animals. There is substantial indirect evidence that insulin resistance may play a role in the etiopathology of hypertension in patients and experimental models (5–8). Furthermore, there is a highly significant relationship between obesity, plasma insulin concentration, and increased BP, although the mechanisms by which insulin resistance, hyperinsulinemia, or both increase the risk of developing cardiovascular disease are not well defined. Otherwise, the prevalence of hypertension in type 2 diabetes mellitus is increased threefold, and the coexistence of hypertension in diabetic patients greatly enhances the development of cardiovascular disease and chronic renal failure (16–22). The link between insulinemia resistance and high BP described above does not prove the presence of causal relationships, even though experimental findings have shown possible mechanisms that may account for a putative relationship. However, taking prior data into account, our findings may suggest that the progression of insulin resistance can cause profound effects on cardiovascular disorder, particularly on the arterial pressure in 8-week HFD-treated and glucose-intolerant animals.

We and others (31–35) have previously shown that oral glucose load and insulin stimulated the kidney sodium potassium adenosine triphosphatase pump and the sodium-hydrogen antiporter, two major renal tubular transports for sodium absorption. Reports indicated that sodium retention, facilitated by hyperinsulinemia, could be an important factor in the pathogenesis of hypertension in insulin resistance (5, 31–34). However, in the current study, we found an unexpected and similar tubular sodium handling, estimated by CLi, in 8-week-old HFD-treated rats and age-matched ND animals. This finding suggests that elevated BP in the present HFD model, at least in part, occurs independently of renal sodium handling dysfunction. Also, this result should raise additional mechanisms to increasing BP in HFD-treated rats, beyond those involved in the renal sodium transport. In this way, as observed in the current study, the impaired insulin stimulated uptake of glucose frequently associated with impaired vasodilatation has been shown to be an early manifestation in insulin-resistant models (5, 19, 31–34). Otherwise, hyperglycemia is believed to cause sympatho-excitation (36–38), which may also contribute to increased BP in HFD-fed rats. In fact, increased calorie intake enhances BP in obese rats by augmentation of the autonomic nerve activation (31, 36). Concomitantly, sympathetic stimulation may enhance insulin resistance (37, 38). Thus, we may hypothesize that sympathetic overactivity and glucose intolerance may stimulate each other, both of which may contribute to a rise in BP. We may not rule out that inter-feeding hyperglycemia with increased glucose glomerular filtered load and sodium-glucose reabsorption may contribute, long-term, to decreased renal sodium excretion and, by association, enhanced BP, as observed in this study. However, changes in the renal nerve activity, renin–angiotensin system, and natriuretic peptide release before or after a glucose load were not tested in the present experiments. Thus, the precise mechanism by which arterial BP enhances in HFD group remains to be elucidated.

Despite unchanged renal function, this study showed a significant higher urinary protein excretion and reduced urinary excretion of magnesium in 8-week HFD-treated rats when compared with appropriated ND group. Recently, studies performed in our laboratory verified beyond 7th week of diet in HFD group, a striking enhancement of the glomerular expression of TGFβ-1, desmin, fibronectin, and collagen, intrinsically related to fibrotic process despite unchanged serum lipids composition compared with control group (18). The significant higher proteinuria found in this study may support these recent findings, indicating that a diet rich in saturated fats is directly associated with the presence of proteinuria in middle-aged adults and elderly subjects (12). Kim et al. (12) proposed the hypothesis that increased renal inflammation markers may be related to saturated fat intake, podocyte effacement, and elevated proteinuria. Recent studies (39, 40) have demonstrated that HFD-treated rats and women with high fat mass index exhibited a spectrum of metabolic abnormalities, the more prominent being dyslipidemia, hyperoxaluria, hypercalciuria, dysproteinuria, low urinary magnesium excretion, loss of bone calcium, and calcium phosphate nephrocalcinosis, results partially shown in the current study. These findings suggest that obesity and fat diet intake may have significantly influence on urine composition in terms of lithogenesis promoters and inhibitors. We suppose that the absence of renal functional modifications in HFD rats, despite higher proteinuria in these animals is related to a lack of time of treatment for detection of these amendments, the differences arising from the rats gender once majority previous studies are made in male rats as well as the inability of the techniques used in this study for detection of renal functional changes

Although the link between glucose intolerance and hypertension described in the present model does not prove the presence of causal relationships, experimental findings have shown possible mechanisms, which may account for a putative relationship. In this way, confirmatory experiments are needed before this conclusion can be wholly made.

## Conclusion

In conclusion, we may state that 8-week female HFD-fed rats compared with ND group stimulate harmful effects, such as BP rise and peripheral glucose intolerance without significant modifications on glomerular filtration rate and tubular sodium handling. Increased BP occurs through insulin resistance and supposedly decreased vasodilatation response. Further studies are needed to better understand the metabolic and cardiovascular changes induced by excessive long-term fat consumption and its implications on the development of the renal disease.
